# Supplementary material for: Direct dependencies between neurons explain activity
Source: ArXiv. 2026 Mar 20:arXiv:2504.08637v3. Preprint. [Version 3] (PMC13015593)
Supplement: Supplement 1 [file NIHPP2504.08637v3-supplement-1.pdf]

Supplementary Information

1. Introduction

In this Supplementary Information, we provide extended analysis and discussion to support the results in the main text. The sections are ordered based on their references in the main text. In Sec. 2, we derive an analytic approximation to the drop in entropy that results from including an additional input in the maximum entropy model; we use this approximation to speed up the greedy algorithm that identifies optimal inputs. In Sec. 3, we demonstrate that our procedure for selecting the complete number of inputs n^* avoids overfitting the activity of the output neuron. In Sec. 4, we show that the central results in the main text hold for the average neuron, not just median. In Sec. 5, we show that the central results hold after downsampling the recordings in time. In Sec. 6, we show that the central results hold across different halves of the recordings. In Sec. 7, we show that direct dependencies predict the complex higher-order dependencies between neurons consistently across different species and neural systems. In Sec. 8, we explore the structures of the inferred weights in different neuronal populations. In Sec. 9, we study direct dependencies in an electrophysiological recording of neuronal spiking in the salamander retina. In Sec. 10, we demonstrate how the maximum entropy model can be generalized to include time-delayed dependencies. Finally in Sec. 11, we demonstrate that the maximum entropy model achieves exact inference in the Ising model, which has long been used as a simplified model of recurrent neuronal activity.

2. Approximate change in entropy

Consider a binary output $y \in \{0,1\}$ and $n-1$ binary inputs $\boldsymbol{x} = \{x_1, \ldots, x_{n-1}\} \in \{0,1\}^{n-1}$. The maximum entropy model consistent with the direct dependencies $P(y|x_i)$, where $i \in \{1, \ldots, n-1\}$, is given by

$$P(y|\boldsymbol{x}) = \frac{1}{Z(\boldsymbol{x})} e^{y\left(b + \sum_{i=1}^{n-1} w_i x_i\right)}, \tag{6}$$

where $Z(\boldsymbol{x}) = 1 + e^{b + \sum_{i=1}^{n-1} w_i x_i}$ ensures normalization. For ease of derivation, we define $x_0 = 1$

and $w_0 = b$, yielding

$$P(y|\boldsymbol{x}) = \frac{1}{Z(\boldsymbol{x})} e^{y \sum_{i=0}^{n-1} w_i x_i}. \tag{7}$$

The entropy of the model takes the form

$$S_{\text{dir}} = \langle \log Z(\boldsymbol{x}) \rangle_{\boldsymbol{x}} - \sum_{i=0}^{n-1} w_i \langle y x_i \rangle, \tag{8}$$

where $\langle \cdot \rangle_{\boldsymbol{x}}$ represents an empirical average over the inputs $\boldsymbol{x}$, and $\langle \cdot \rangle$ represents an empirical average over the inputs and the output y.

We seek an analytic approximation for the drop in entropy ΔS_{dir} after including a new input x_n in the model. Using perturbation theory, we can expand ΔS_{dir} in the limit of a small prediction error $\langle y x_n \rangle - \langle y x_n \rangle_P$, where $\langle y x_n \rangle_P = \langle \sum_y y x_n P(y|\boldsymbol{x}) \rangle_{\boldsymbol{x}}$ is the correlation predicted by the existing model (without x_n as an input, or, equivalently, with $w_n = 0$). To second order, we have

$$\Delta S_{\text{dir}} = (\langle y x_n \rangle - \langle y x_n \rangle_P) \left. \frac{dS_{\text{dir}}}{d\langle y x_n \rangle_P} \right|_{w_n=0} + \frac{1}{2} (\langle y x_n \rangle - \langle y x_n \rangle_P)^2 \left. \frac{d^2 S_{\text{dir}}}{d\langle y x_n \rangle_P^2} \right|_{w_n=0}. \tag{9}$$

The first derivative takes the form

$$\frac{dS_{\text{dir}}}{d\langle y x_n \rangle_P} = \frac{d\langle \log Z(\boldsymbol{x}) \rangle_{\boldsymbol{x}}}{d\langle y x_n \rangle_P} - w_n - \sum_{i=0}^{n-1} \left(\langle y x_i \rangle \frac{dw_i}{d\langle y x_n \rangle_P} + w_i \frac{d\langle y x_i \rangle}{d\langle y x_n \rangle_P} \right). \tag{10}$$

From the maximum entropy constraints, we know that $\frac{d\langle y x_i \rangle}{d\langle y x_n \rangle_P} = 0$. We also have

$$\frac{d\langle \log Z(\boldsymbol{x}) \rangle_{\boldsymbol{x}}}{d\langle y x_n \rangle_P} = \sum_{i=0}^{n-1} \frac{\partial \langle \log Z(\boldsymbol{x}) \rangle_{\boldsymbol{x}}}{\partial w_i} \frac{dw_i}{d\langle y x_n \rangle_P} = \sum_{i=0}^{n-1} \langle y x_i \rangle_P \frac{dw_i}{d\langle y x_n \rangle_P} = \sum_{i=0}^{n-1} \langle y x_i \rangle \frac{dw_i}{d\langle y x_n \rangle_P}. \tag{11}$$

Plugging into Eq. (10), we have

$$\frac{dS_{\text{dir}}}{d\langle y x_n \rangle_P} = -w_n. \tag{12}$$

Thus, to first order, ΔS_{dir} vanishes.

The second derivative is given by

$$\frac{d^2 S_{\text{dir}}}{d\langle y x_n \rangle_P^2} = -\frac{dw_n}{d\langle y x_n \rangle_P} = -\left(\frac{d\langle y x_n \rangle_P}{dw_n} \right)^{-1}. \tag{13}$$

We have

$$\frac{d\langle yx_n\rangle_P}{dw_n} = \frac{\partial\langle yx_n\rangle_P}{\partial w_n} + \sum_{i=0}^{n-1} \frac{\partial\langle yx_n\rangle_P}{\partial w_i}\frac{dw_i}{dw_n}. \tag{14}$$

The derivative $\frac{dw_i}{dw_n}$ arises from the fact that, as w_n changes, the existing weights w_i must change to maintain the maximum entropy constraints $\langle yx_i\rangle_P = \langle yx_i\rangle$. Thus, we have

$$0 = \frac{d\langle yx_i\rangle_P}{dw_n} = \frac{\partial\langle yx_i\rangle_P}{\partial w_n} + \sum_{j=0}^{n-1} \frac{\partial\langle yx_i\rangle_P}{\partial w_j}\frac{dw_j}{dw_n}. \tag{15}$$

For $i, j \in \{0, \ldots, n-1\}$, we define the matrix

$$M_{ij} = \frac{\partial\langle yx_i\rangle_P}{\partial w_j} \tag{16}$$

$$= \left\langle \frac{\partial}{\partial w_j}\frac{1}{Z(\boldsymbol{x})}\sum_y yx_i e^{y\sum_{k=0}^{n-1} w_k x_k} \right\rangle_{\boldsymbol{x}} \tag{17}$$

$$= \left\langle \frac{1}{Z(\boldsymbol{x})}\sum_y y^2 x_i x_j e^{y\sum_{k=0}^{n-1} w_k x_k} - \frac{1}{Z(\boldsymbol{x})^2}\left(\sum_y yx_i e^{y\sum_{k=0}^{n-1} w_k x_k}\right)\left(\sum_{y'} y' x_j e^{y'\sum_{k=0}^{n-1} w_k x_k}\right)\right\rangle_{\boldsymbol{x}} \tag{18}$$

$$= \langle yx_ix_j\rangle_P - \langle yy'x_ix_j\rangle_P. \tag{19}$$

Similarly, for $i \in \{0, \ldots, n\}$, we have

$$\frac{\partial\langle yx_i\rangle_P}{\partial w_n} = \langle yx_ix_n\rangle_P - \langle yy'x_ix_n\rangle_P. \tag{20}$$

These definitions yield

$$\frac{dw_i}{dw_n} = -\sum_{j=0}^{n-1}(M^{-1})_{ij}\frac{\partial\langle yx_j\rangle_P}{\partial w_n}. \tag{21}$$

Plugging into Eqs. (13-14), we have

$$\frac{d^2 S_{\text{dir}}}{d\langle yx_n\rangle_P^2} = -\left(\frac{d\langle yx_n\rangle_P}{dw_n}\right)^{-1} = -\left(\frac{\partial\langle yx_n\rangle_P}{\partial w_n} - \sum_{i,j=0}^{n-1}\frac{\partial\langle yx_i\rangle_P}{\partial w_n}(M^{-1})_{ij}\frac{\partial\langle yx_j\rangle_P}{\partial w_n}\right)^{-1} \tag{22}$$

We therefore arrive at an analytic approximation to the change in entropy

$$\Delta S_{\text{dir}} = -\frac{1}{2}\frac{(\langle yx_n\rangle - \langle yx_n\rangle_P)^2}{\frac{\partial\langle yx_n\rangle_P}{\partial w_n} - \sum_{i,j=0}^{n-1}\frac{\partial\langle yx_i\rangle_P}{\partial w_n}(M^{-1})_{ij}\frac{\partial\langle yx_j\rangle_P}{\partial w_n}}. \tag{23}$$

Note that this change is always negative, which follows from the fact that the maximum entropy must decrease with increasing constraints.

3. Restricting inputs avoids overfitting

When modeling the output of a neuron as a function of inputs using Eq. (6), we need to make sure we are not overfitting the data, thus leading to an artificially low entropy S_{dir}. For a given output neuron, we use a greedy algorithm to iteratively select the n optimal inputs that provide the best description of the output. Each time we add a new input x_i to the model, we include an additional constraint on the direct dependence $P(y|x_i)$, which leads to a lower entropy S_{dir}. In large populations, such as those in the hippocampus and visual cortex studied in the main text, if we include all possible inputs to a given output neuron, then the entropy S_{dir} drops to zero (Fig. 3 in the main text), and we have likely overfit the data.

To avoid overfitting, we terminate the greedy algorithm when the model is able to predict the direct dependencies $P(y|x_i)$ for all neurons x_i that are not included as inputs in the model (within experimental errors). This ensures that we do not constrain any dependencies that the model already predicts, which would amount to fitting statistical noise. Note that we only consider neurons with positive correlations $\langle yx_i\rangle > 0$, such that the dependencies $P(y|x_i)$ are well-defined. Upon termination of the greedy algorithm, we arrive at a model (which we refer to as "complete") with n^* inputs that captures the direct dependencies $P(y|x_i)$ on all other neurons in the population (with $\langle yx_i\rangle > 0$), either by fitting or prediction.

Here, we demonstrate that by restricting the number of inputs we avoid overfitting. For each of the four recordings analyzed in the main text, we randomly divide the samples of activity into a training set (90%) and test set (10%). For each neuron, we infer the minimal computations in the training data as we increase the number of optimal inputs n. We then compute the negative log-likelihood of each model in both the training and test data,

$$\ell = -\langle \log P(y|\boldsymbol{x})\rangle_{\text{train}} \qquad \text{and} \qquad \ell_{\text{test}} = -\langle \log P(y|\boldsymbol{x})\rangle_{\text{test}}, \tag{24}$$

where $\langle\cdot\rangle_{\text{train}}$ and $\langle\cdot\rangle_{\text{test}}$ represent empirical averages over the training and test data, respectively.

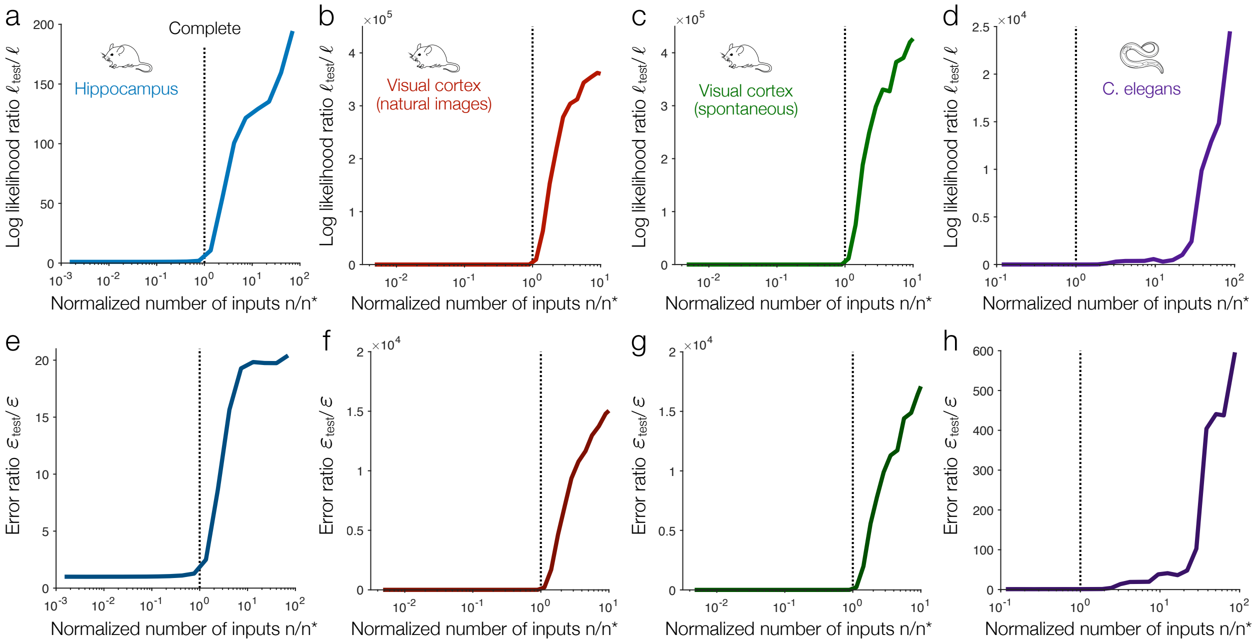

Fig. S1 | Training and test errors. a-d, Ratio of test and training log-likelihoods ℓ_{test}/ℓ versus the number of inputs n normalized by n^* for populations of neurons in the mouse hippocampus (**a**),[11] mouse visual cortex during responses to natural images (**b**) and spontaneous activity (**c**),[10] and the brain of *C. elegans* (**d**).[12] Dashed lines indicate the complete model with $n = n^*$. **e-h**, Ratio of test and training errors $\varepsilon_{\text{test}}/\varepsilon$ versus the number of inputs n normalized by n^* for the same data as **a-d**. Values in the hippocampus are averaged over all $N = 1485$ neurons in the population (**a** and **e**); values in the visual cortex are averaged over 100 randomly-selected neurons among a population of $N = 11,445$ (**b, c, f**, and **g**); and values in *C. elegans* are averaged over all $N = 128$ neurons in the population (**d** and **h**).

For the hippocampus and visual cortex, as we increase the number of inputs n, we see that the ratio ℓ_{test}/ℓ remains small until we reach n^*; for $n > n^*$, the model begins fitting dependencies $P(y|x_i)$ that it can already predict, and ℓ_{test} increases dramatically relative to ℓ (Fig. S1a-c). For *C. elegans*, we see that the log-likelihood ratio ℓ_{test}/ℓ doesn't increase until $n \gtrsim 10n^*$, which indicates that one might be able to increase the number of inputs n^* in the complete model without overfitting (Fig. S1d).

We also consider the model errors directly,

$$\varepsilon = \langle 1 - P(y|\boldsymbol{x}) \rangle_{\text{train}} \qquad \text{and} \qquad \varepsilon_{\text{test}} = \langle 1 - P(y|\boldsymbol{x}) \rangle_{\text{test}}. \tag{25}$$

Just as for the log-likelihoods, across all populations, we find that the ratio $\varepsilon_{\text{test}}/\varepsilon$ remains small for $n \leq n^*$ (Fig. S1e-h). In the hippocampus and visual cortex, the test errors increase sharply for $n > n^*$, indicating overfitting (Fig. S1e-g); while in *C. elegans*, the test errors don't increase significantly until $n \gtrsim 10n^*$ (Fig. S1h). Together, these results demonstrate that, by limiting the number of inputs to n^*, the complete models avoid overfitting the data. In turn, this tells us that the low entropies S_{dir} of the complete models are not due to overfitting (Fig. 3f in the main text); instead, they indicate that the majority of neuronal variability is explained by direct dependencies on relatively small numbers of inputs.

4. Average variability explained by direct dependencies

In the main text, most quantities are provided as medians and interquartile ranges across neurons in each population. In Fig. S2, we show that the central results in the main text (Fig. 3) also hold for the average neuron. Specifically, if inputs are chosen optimally, we find that the direct entropy S_{dir} drops sharply as a function of the number of inputs n (Fig. S2a-d). Meanwhile, for the same numbers of random inputs, direct dependencies explain much less of the variability in activity. On average across the hippocampal neurons, we find that the complete models only require $n^* = 258$ inputs to capture all $N - 1 = 1484$ of the direct dependencies in the population (Fig. S2e) and explain $1 - S_{\text{dir}}/S_{\text{tot}} \approx 90\%$ of the variability in activity (Fig. S2f). On average across 100 cortical neurons, complete models only require $n^* = 121$ inputs to capture $N - 1 = 11,444$ direct dependencies and explain 92% of the total entropy; these numbers are consistent between spontaneous activity and responses to natural images. Finally, on average across the *C. elegans* brain, complete models only require $n^* = 6$ inputs to capture $N - 1 = 127$ direct dependencies and explain 59% of the total entropy.

5. Downsampling in time

For each of the recordings in the main text, we study activity on the fastest possible timescale, which is determined by the sample period Δt of the experiment itself. One could instead study activity on longer timescales by downsampling the recording in time. To investigate the effect of

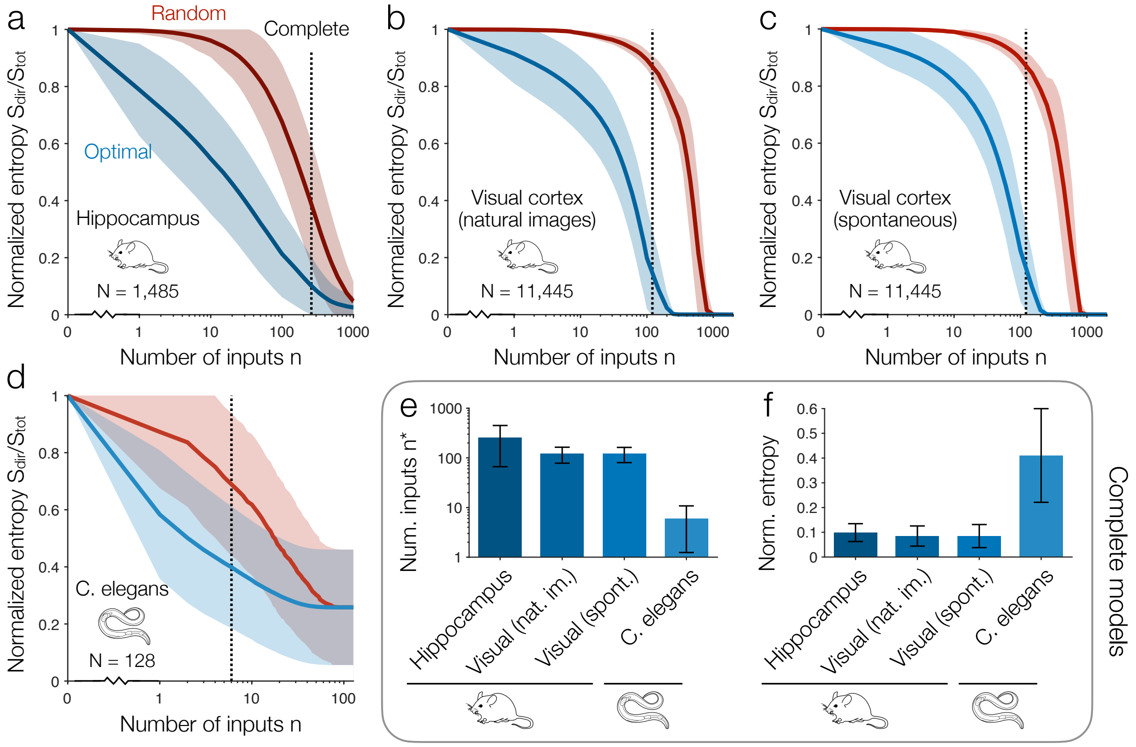

Fig. S2 | **Average variability explained by direct dependencies.** **a**, Direct entropy S_{dir} normalized by total entropy S_{tot} for n inputs chosen optimally (blue) or randomly (red). Lines and shaded regions represent means and one-standard-deviation error bars across all $N = 1485$ hippocampal neurons.[11,41] Dashed line indicates the average minimal number of inputs n^* needed to capture all the direct dependencies. **b-d**, Normalized model entropy $S_{\text{dir}}/S_{\text{tot}}$ versus number of inputs n for 100 random output neurons within a population of $N = 11,445$ cells in the mouse visual cortex during responses to natural images (**b**) and spontaneous activity (**c**),[10] and for $N = 128$ neurons in the brain of *C. elegans* (**d**).[12] **e-f**, For the complete models in **a-d**, we compare the minimal number of inputs n^* needed to capture all direct dependencies (**e**) and the normalized entropies $S_{\text{dir}}/S_{\text{tot}}$ (**f**). Values and error bars represent means and standard deviations across neurons.

downsampling, we consider the hippocampal population, which was recorded with the shortest period $\Delta t = 1/30\,\text{s}$. After randomly selecting one out of every m samples, we compute the total entropy S_{tot} and the entropy of the complete maximum entropy model S_{dir} for each neuron with an effective time resolution of $\Delta\tilde{t} = m\Delta t$. As we increase the sample period $\Delta\tilde{t}$ up to one second, we find that S_{tot}, S_{dir}, and the normalized entropy $S_{\text{dir}}/S_{\text{tot}}$ all remain constant (Fig. S3). This indicates that our results are robust to downsampling in time.

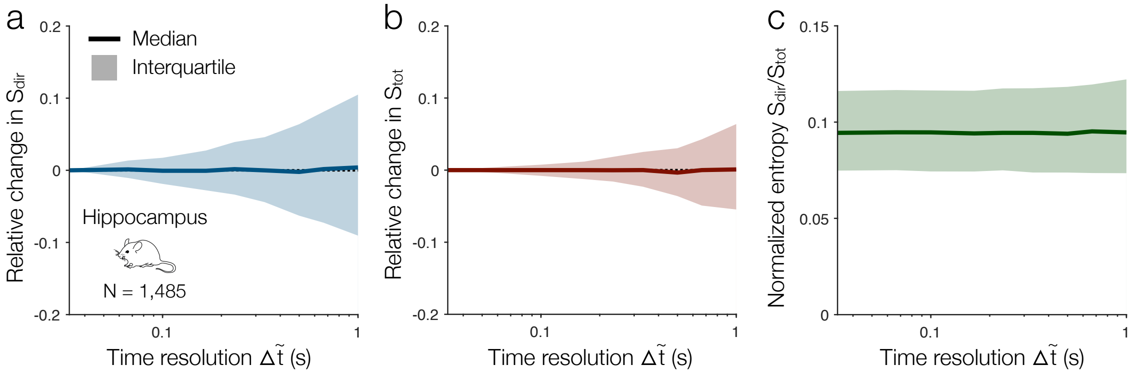

Fig. S3 | **Consistency under downsampling. a-c**, Relative change in direct entropy S_{dir} (**a**), relative change in total entropy S_{tot} (**b**), and change in normalized entropy $S_{\text{dir}}/S_{\text{tot}}$ (**c**) as functions of time resolution $\Delta\tilde{t}$ after downsampling. Lines and shaded regions represent medians and interquartile ranges across all $N = 1485$ hippocampal neurons.[11,41] Direct entropies S_{dir} are computed using the complete maximum entropy models fit to the full data with empirical averages taken over subsamples of the input activity.

6. Consistency over time

In addition to the effects of downsampling, one can also study whether our results change over time. To do so, we divide each recording into halves and re-fit every model based on the statistics measured in each half of the data. We find that the direct entropy S_{dir} of the complete models remains consistent between the first and second halves of each recording (Fig. S4a). Similarly, the normalized entropy $S_{\text{dir}}/S_{\text{tot}}$ (and therefore the fraction of entropy explained by direct dependencies $1 - S_{\text{dir}}/S_{\text{tot}}$) also remains consistent over time (Fig. S4b). This indicates that our central results do not change significantly over the length of each recording.

7. Predicting higher-order dependencies

Given the direct dependencies $P(y|x_i)$, the model in Eq. (6) is maximally random with regard to all complex higher-order dependencies. Thus, if the model is capable of predicting the higher-order dependencies on two, three, or more inputs, then these can be viewed as emerging naturally from simpler direct dependencies. As discussed in the main text, predicting a given k^{th}-order dependence $P(y|x_1, \ldots, x_k)$ is equivalent to predicting the correlations between the output y and

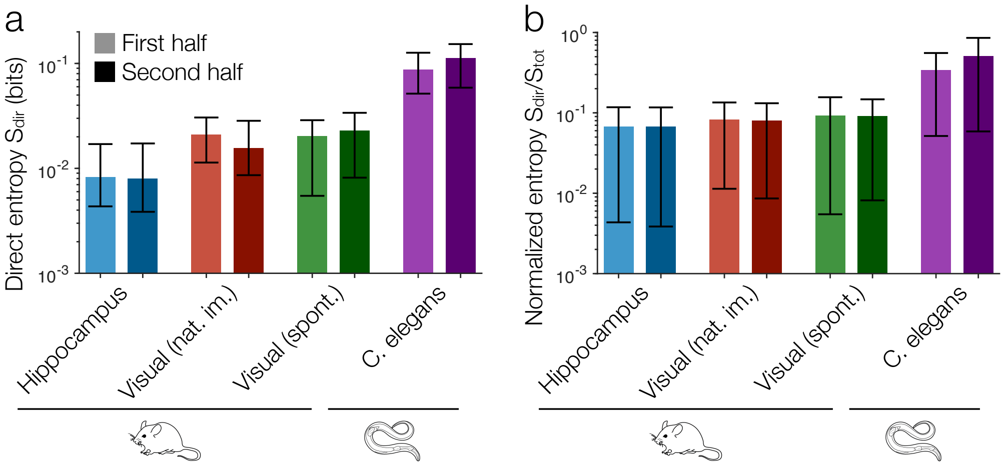

Fig. S4 | **Consistency between halves of each recording. a-b**, Comparison of the direct entropies of the complete models S_{dir} (**a**) and normalized entropies $S_{\text{dir}}/S_{\text{tot}}$ (**b**) between halves of each recording. Values and error bars represent medians and interquartile ranges across neurons.

all subsets of the k inputs. Thus, if the model captures all of the $(k-1)^{\text{th}}$-order dependencies, all that remains is the $(k+1)^{\text{th}}$-order correlation $\langle yx_1 \cdots x_k \rangle$.

As discussed above, for each output neuron y, we use our greedy algorithm to identify the smallest number of inputs n^* for which the model captures all of the positive correlations $\langle yx_i \rangle > 0$ in the population; this defines our complete model. Across each of the populations, we confirm that our complete models match all of the direct correlations within experimental errors (Fig. S5a-d) and, therefore, capture all of the direct dependencies $P(y|x_i)$.

Thus, to predict the second-order dependencies $P(y|x_i, x_j)$, we need only predict the triplet correlations $\langle yx_i x_j \rangle$. In Fig. S5i-l, we show that our models predict nearly all of the triplet correlations within experimental errors. Moreover, the minimal computations correctly predict an even larger proportion of the quadruplet (Fig. S5i-l) and quintuplet (Fig. S5m-p) correlations. Together, these results demonstrate that our minimal computation is capable of predicting the higher-order dependencies on combinations of two, three, or four inputs. In turn, this indicates that the vast majority of higher-order dependencies are explained simple direct dependencies, rather relying on complex interactions between inputs.

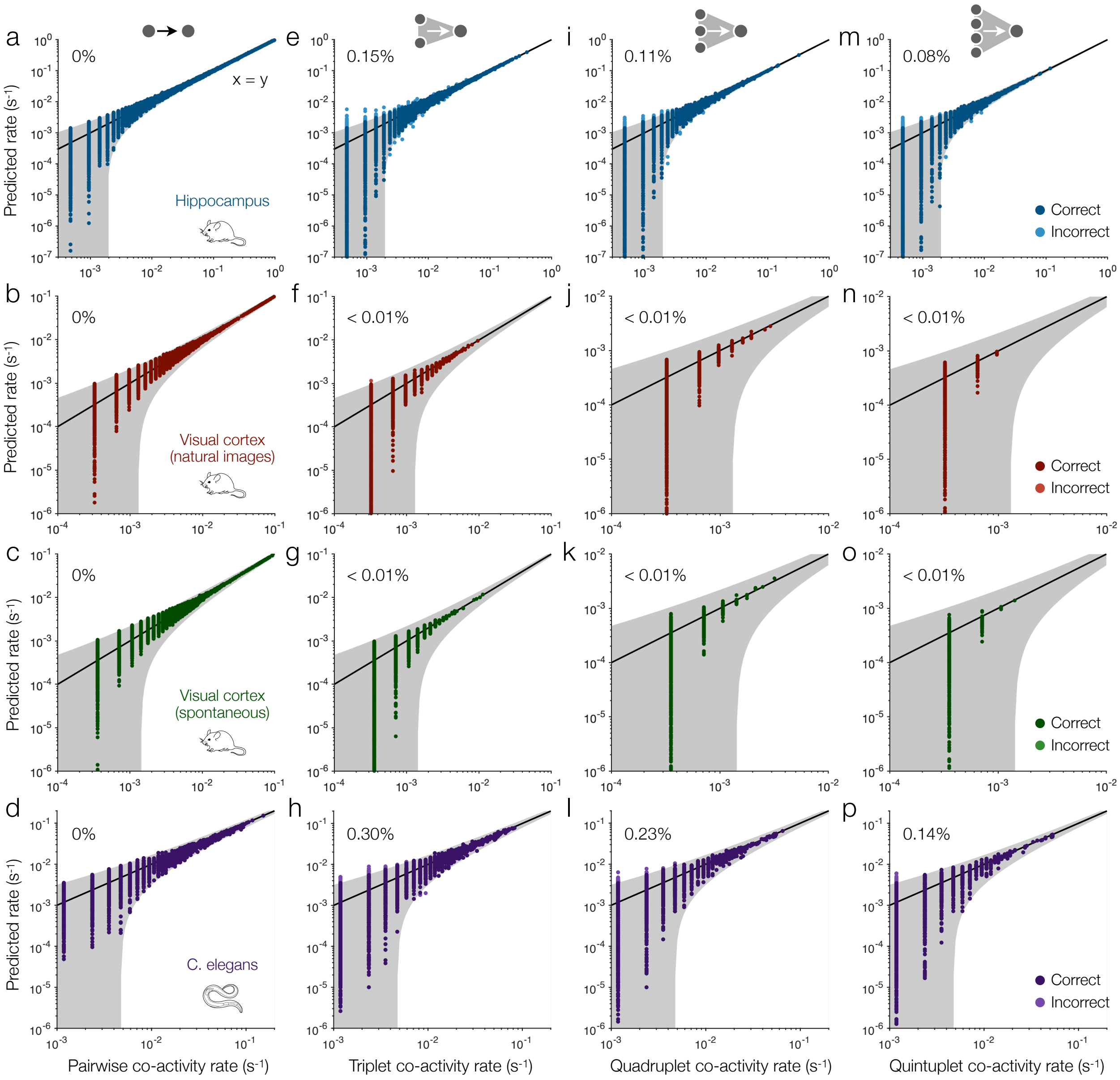

Fig. S5 | **Predicting higher-order correlations. a-d**, Pairwise co-activity rates $\langle yx_i \rangle / \Delta t$ predicted by complete models versus those in the data for populations of neurons in the mouse hippocampus (**a**),[11] mouse visual cortex during responses to natural images (**b**) and spontaneous activity (**c**),[10] and the brain of *C. elegans* (**d**).[12] Lines illustrate equality, shaded regions represent two standard deviations of experimental errors, dark points indicate correct predictions (within errors), light points indicate incorrect predictions, and percentages define the proportion of incorrect predictions. By the definition of the complete models, they correctly capture all pairwise co-activity rates, either by fitting or prediction. **e-p**, Triplet co-activity rates $\langle yx_ix_j \rangle / \Delta t$ (**e-h**), quadruplet co-activity rates $\langle yx_ix_jx_k \rangle / \Delta t$ (**i-l**), and quintuplet co-activity rates $\langle yx_ix_jx_kx_\ell \rangle / \Delta t$ (**m-p**) predicted by complete models versus those in the data for the

same populations as **a-d**. For the mouse hippocampus (blue) and *C. elegans* (purple) populations, we consider all $N = 1485$ and $N = 128$ neurons as outputs, respectively. For the mouse visual cortex (red and green), we consider 100 random output neurons within the population of $N = 11,445$ cells. For the pairwise co-activity rates (**a-d**), for each output neuron y we consider all positive co-activities in the populations. For the higher-order co-activity rates (**e-p**), for each output neuron we consider 100 randomly-selected positive co-activities in the hippocampal (blue) and *C. elegans* (purple) populations and 10^4 randomly-selected positive co-activities in the visual cortex recordings (red and green).

8. Model structures

After constructing the complete model for each output neuron, we have the opportunity to investigate the properties of the inferred weights. In the main text, we show in the mouse hippocampus that the inferred input weights w_i exhibit four key features. First, the weights are sparse, with only a small number of inputs n^* needed to explain all of a neuron's pairwise dependencies (Fig. 3e in the main text). Second, the distribution of magnitudes is heavy-tailed (specifically log-normal), with some rare weights that are orders of magnitude stronger than average (Fig. S6a). Third, the weights are evenly split between positive and negative, suggesting a delicate balance between excitatory and inhibitory interactions (Fig. S6a). Finally, unlike many existing maximum entropy models,[16,36,41] the weights are highly directed, with the connection strength from input i to output j differing significantly from the reverse. These sparse, heavy-tailed, balanced, and directed weights are universal features of synaptic connectivity observed across brain regions and species.[2,3,58–61]

For recordings in the mouse visual cortex and *C. elegans*, we have already seen that the connectivity is sparse (Fig. 3e in the main text), with the complete models requiring an even smaller number of inputs n^* than in the mouse hippocampus. In Fig. S6b-d, we also confirm that the inferred weights w_i are close to log-normally distributed (i.e., heavy-tailed) and quite evenly split between positive and negative (i.e., balanced). Since the weights are also directed, we find that the inferred connectivities share the same four key features across each of the recordings.

In the hippocampal population, we saw in the main text that positive (negative) input weights

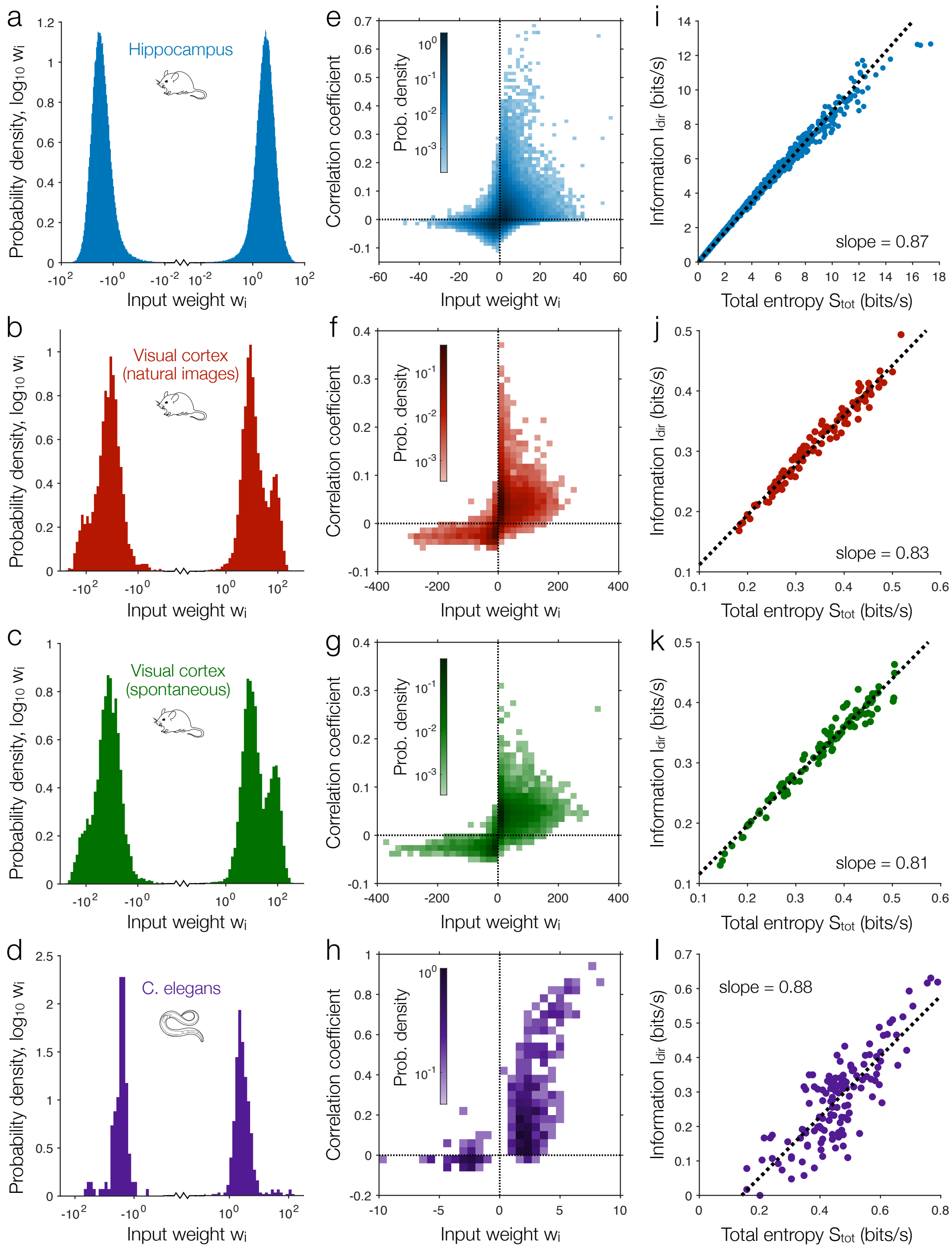

Fig. S6 | **Structure of complete models. a-d**, Distribution of inferred input weights w_i over all complete models in populations in the mouse hippocampus (**a**),[11] mouse visual cortex during responses to natural images (**b**) and spontaneous activity (**c**),[10] and the brain of *C. elegans* (**d**).[12] **e-h**, Probability density of the correlation coefficient and corresponding input weight w_i over all input-output pairs in complete models for the same populations as **a-d**. **i-l**, Information I_{dir} in complete models versus total entropy

S_{tot} (normalized by Δt) across all output neurons; dashed line indicates linear fit. For the mouse hippocampus (blue) and *C. elegans* (purple), we consider all $N = 1485$ and $N = 128$ neurons as outputs, respectively. For the mouse visual cortex (red and green), we consider 100 random output neurons within the population of $N = 11,445$ cells.

w_i tend to produce positive (negative) correlations between the inputs and output in each model (Fig. S6e). In Fig. S6f-h, we observe the same relationship in the mouse visual cortex and *C. elegans* recordings. Finally, we consider the mutual information $I_{\text{dir}} = S_{\text{tot}} - S_{\text{dir}}$ between the inputs and output in the complete models, which provides a tight upper bound on the true mutual information $I_{\text{true}} = S_{\text{tot}} - S_{\text{true}}$. In the hippocampus, we find that the mutual information increases linearly with the total entropy S_{tot}, with each bit generated by a neuron encoding a consistent 0.87 bits of information about its inputs (Fig. S6i). We confirm similar linear relationships across the mouse visual cortex and *C. elegans* recordings (Fig. S6j-l). Together, these results suggest that the amount of information encoded in statistical dependencies may be strikingly consistent across species and neural systems.

9. Electrophysiological activity

By studying the statistical dependencies between neurons, we hope to capture aspects of the true underlying interactions. To have any hope of measuring the output of a neuron and many of its inputs simultaneously, we study recordings that meet three criteria. First, the recordings must be large. In *C. elegans*, each neuron receives synaptic inputs from tens of others;[1] in the mouse cortex, this number increases to thousands or tens of thousands.[3] We therefore focus on recordings that are at least this large. Second, we focus on neural systems with recurrent connectivity, such that each neuron within a recording may receive inputs from the others. Finally, we focus on populations that are spatially contiguous, which ensures that each pair of neurons is physically close and therefore has an opportunity to interact synaptically.

Current experiments that meet these criteria involve calcium imaging of large neuronal populations, as investigated in the main text.[10–12] Most electrophysiological recordings, by contrast,

fail to meet at least one of the above criteria. For example, Neuropixel probes provide the largest electrophysiological recordings involving up to thousands of neurons.[62,65] However, each probe records a spatially elongated one-dimensional slice through the brain, and large-scale recordings involve multiple probes distributed discontiguously throughout a single brain region or multiple regions.

Despite these experimental limitations, our framework can immediately be applied to study electrophysiological recordings. To demonstrate this generality, we investigate the activity of $N = 160$ ganglion cells in the salamander retina recorded using a multi-electrode array in previous experiments.[71] The electrical activity of each cell is binarized within time windows of width $\Delta t = 20\,\text{ms}$. For each neuron, we use our greedy algorithm to identify the optimal inputs. As the number of inputs n increases, we find that the direct entropy S_{dir} decreases exponentially before eventually plateauing at around 70% of the total entropy S_{tot} (Fig. S7a). For random inputs, the direct entropy decreases much more slowly (Fig. S7a). For the median neuron, one requires $n^* = 100$ inputs to explain all $N - 1 = 159$ of the direct dependencies (Fig. S7b, *bottom*). These complete models explain $1 - S_{\text{dir}}/S_{\text{tot}} \approx 30\%$ of the total variability in activity (Fig. S7b, *top*). We note that this relatively low amount of variability explained by direct dependencies is not surprising given that retinal ganglion cells receive most of their information from photoreceptors. We therefore expect most of the variability in activity to arise from latent variables (Fig. 1d in the main text). Indeed, relative to the recordings analyzed in the main text, direct dependencies fail to predict a larger proportion of higher-order dependencies on multiple inputs (Fig. S7c) as well as time-delayed dependencies on past inputs (Fig. S7d). As electrophysiological recordings advance to record larger contiguous populations, our maximum entropy framework provides a foundation for studying direct dependencies in future work.

10. Time-delayed dependencies

In the main text, we demonstrate that the vast majority of time-delayed dependencies are explained by instantaneous direct dependencies (Fig. 4h-n in the main text). However, a small number of significant time-delayed dependencies remain unexplained. To illustrate how these

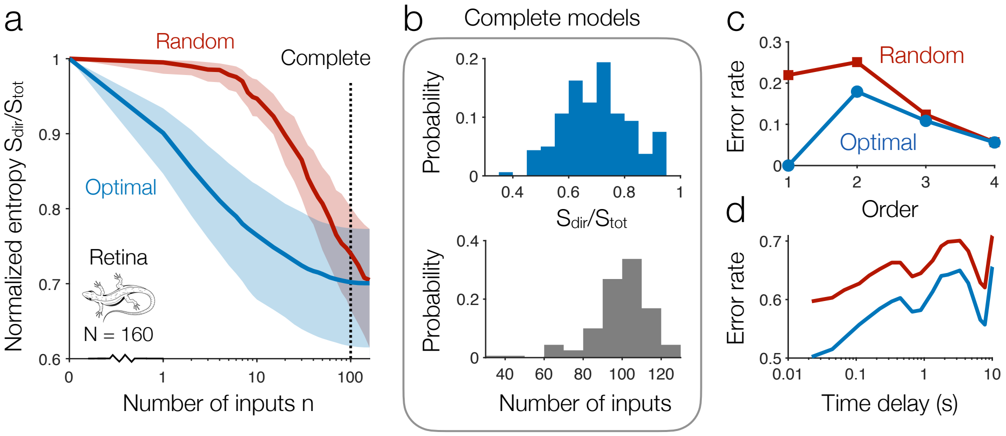

Fig. S7 | Electrophysiological recording of retinal ganglion cells. a, Direct entropy S_{dir} normalized by total entropy S_{tot} for n inputs chosen optimally (blue) or randomly (red). Lines and shaded regions represent medians and interquartile ranges across all $N = 160$ neurons within an electrophysiological recording of ganglion cells in the salamander retina.[71] Dashed line indicates the minimal number of inputs n^* needed to capture all the direct dependencies for the median neuron. **b**, Distributions of normalized entropies $S_{\text{dir}}/S_{\text{tot}}$ (*top*) and inputs n^* (*bottom*) for complete models over different retinal ganglion neurons. **c-d**, Fractions of higher-order dependencies and time-delayed dependencies not predicted by direct dependencies.

time-delayed dependencies can be incorporated into our maximum entropy framework, we investigate a simulated population of $N = 100$ neurons with known temporal interactions. For each neuron i, we draw time-delayed input weights $W_{ij}(\delta t)$ for every other neuron j from a zero-mean Gaussian with standard deviation

$$\sigma(\delta t, \tau) = \frac{e^{-\delta t/\tau}}{\sum_{\delta t'=0}^{T} e^{-\delta t'/\tau}}, \tag{26}$$

where $\delta t = 0, \ldots, T$ is the time-delay, T is the length of the longest time-delayed interaction, and τ is a time constant that defines how quickly the interactions decay. We simulate the population dynamics based on the logistic model

$$P(x_i(t) = 1 \,|\, \boldsymbol{x}(t), \ldots, \boldsymbol{x}(t-T)) = \sigma\left[\sum_{\delta t=0}^{T} \sum_{j \neq i} W_{ij}(\delta t) x_j(t - \delta t)\right], \tag{27}$$

where $\sigma(\cdot)$ is the sigmoid function and the vector $\boldsymbol{x}(t) = \{x_i(t)\}$ defines the activity of all neurons at time t.

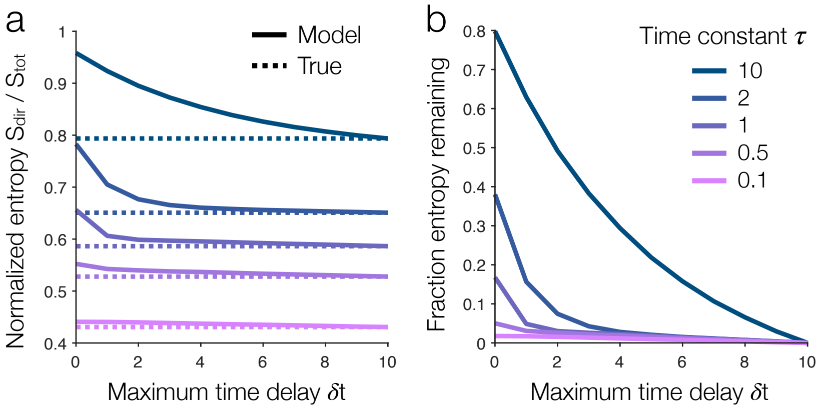

Fig. S8 | Maximum entropy models with time-delayed dependencies. **a**, Normalized entropy $S_{\text{dir}}/S_{\text{tot}}$ for maximum entropy models constrained to match direct time-delayed dependencies $P(x_i(t)|x_j(t-\delta t))$ up to a maximum delay δt. Dashed lines indicate the normalized entropy $S_{\text{true}}/S_{\text{tot}}$ for the ground-truth models, which are defined in Eq. (27) with a maximum time delay $T = 10$. Colors reflect different time constants τ. **b**, Fraction of available entropy not explained by maximum entropy models $(S_{\text{dir}} - S_{\text{true}})/(S_{\text{tot}} - S_{\text{true}})$ versus maximum time delay. In both panels, dynamics are simulated for $N = 100$ neurons and values are averages over all neurons.

Given simulated dynamics, we can construct the maximum entropy model consistent with not only instantaneous dependencies $P(x_i(t)|x_j(t))$, but also time-delayed dependencies $P(x_i(t)|x_j(t-\delta t))$. As we include more of the time-delayed dependencies in the maximum entropy model, the normalized entropy $S_{\text{dir}}/S_{\text{tot}}$ quickly plateaus to the true value $S_{\text{true}}/S_{\text{tot}}$ of the ground-truth model (Fig. S8a). As expected, this convergence is faster for dynamics in which the time-delayed interactions $w_{ij}(\delta t)$ decay more quickly (that is, for smaller time constants τ; Fig. S8b). For $\tau = 1$, instantaneous dependencies explain $(S_{\text{tot}} - S_{\text{dir}})/(S_{\text{tot}} - S_{\text{true}}) \approx 83\%$ of the available variance, and this fraction increases to 98% for $\tau = 0.1$. These results demonstrate that direct dependencies may be surprisingly effective in capturing time-delayed interactions.

11. Exact inference in Ising model

We have seen that the maximum entropy model in Eq. (6) provides a tight approximation to the activity of real neurons. But how does the model perform in an artificial system where the underlying interactions are known? As a salient example, we consider Ising models, which are equivalent to Hopfield networks (with non-zero temperature),[5] Boltzmann machines,[66] and

maximum entropy models for joint activity.[16,36,37,41] In each of these forms, the Ising model has provided key insights into neural computation, both in the brain and artificial networks. Here, we show that the maximum entropy model provides exact inference in the Ising model.

Consider a network with N binary neurons defined by the state vector $\boldsymbol{x} = \{x_1, \ldots, x_N\}$. Each neuron has a bias b_i that influences it toward activity or silence, and neurons interact via a symmetric interaction matrix $W_{ij} = W_{ji}$. The joint probability of a given activity state is defined by the Boltzmann distribution

$$P(\boldsymbol{x}) = \frac{1}{Z} e^{\sum_i b_i x_i + \frac{1}{2}\sum_{ij} W_{ij} x_i x_j}, \tag{28}$$

where

$$Z = \sum_{\boldsymbol{x} \in \{0,1\}^N} e^{\sum_i b_i x_i + \frac{1}{2}\sum_{ij} W_{ij} x_i x_j} \tag{29}$$

is the partition function, which ensures normalization.

Suppose we want to study the output of one neuron (say x_i) in response to the remaining neurons $\boldsymbol{x}_{-i} = \{x_1, \ldots, x_{i-1}, x_{i+1}, \ldots, x_N\}$. Specifically, we would like to compute the conditional probability $P(x_i|\boldsymbol{x}_{-i}) = P(\boldsymbol{x})/P(\boldsymbol{x}_{-i})$, where

$$P(\boldsymbol{x}_{-i}) = \sum_{x_i = 0,1} P(\boldsymbol{x}) = \frac{1}{Z} e^{\sum_{j \neq i} b_j x_j + \frac{1}{2}\sum_{jk \neq i} W_{ij} x_i x_j} \left(1 + e^{b_i + \sum_j W_{ij} x_j}\right). \tag{30}$$

We therefore have

$$P(x_i|\boldsymbol{x}_{-i}) = \frac{e^{x_i(b_i + \sum_j W_{ij} x_j)}}{1 + e^{b_i + \sum_j W_{ij} x_j}} = \sigma\big(b_i + \sum_j W_{ij} x_j\big). \tag{31}$$

This tells us that the conditional probability of one Ising variable in response to the rest of the system takes precisely the same form as the model in Eq. (6). Thus, given the direct dependencies $P(x_i|x_j)$ for all $j \neq i$, the maximum entropy model is guaranteed to recover to the correct bias b_i and weights W_{ij}. This approach provides an efficient method for inference in high-dimensional Ising models.[72]
